# Semantic Features Reveal Different Networks During Word Processing: An EEG Source Localization Study

**DOI:** 10.3389/fnhum.2018.00503

**Published:** 2018-12-13

**Authors:** Mansoureh Fahimi Hnazaee, Elvira Khachatryan, Marc M. Van Hulle

**Affiliations:** Laboratory for Neuro- and Psychophysiology, Department of Neurosciences, KU Leuven, Leuven, Belgium

**Keywords:** high-density EEG, source localization, semantic category representation, single word processing, linear mixed effect model

## Abstract

The neural principles behind semantic category representation are still under debate. Dominant theories mostly focus on distinguishing concrete from abstract concepts but, in such theories, divisions into categories of concrete concepts are more developed than for their abstract counterparts. An encompassing theory on semantic category representation could be within reach when charting the semantic attributes that are capable of describing both concept types. A good candidate are the three semantic dimensions defined by Osgood (potency, valence, arousal). However, to show to what extent they affect semantic processing, specific neuroimaging tools are required. Electroencephalography (EEG) is on par with the temporal resolution of cognitive behavior and source reconstruction. Using high-density set-ups, it is able to yield a spatial resolution in the scale of millimeters, sufficient to identify anatomical brain parcellations that could differentially contribute to semantic category representation. Cognitive neuroscientists traditionally focus on scalp domain analysis and turn to source reconstruction when an effect in the scalp domain has been detected. Traditional methods will potentially miss out on the fine-grained effects of semantic features as they are possibly obscured by the mixing of source activity due to volume conduction. For this reason, we have developed a mass-univariate analysis in the source domain using a mixed linear effect model. Our analyses reveal distinct networks of sources for different semantic features that are active during different stages of lexico-semantic processing of single words. With our method we identified differences in the spatio-temporal activation patterns of abstract and concrete words, high and low potency words, high and low valence words, and high and low arousal words, and in this way shed light on how word categories are represented in the brain.

## Introduction

Different cortical areas are involved in representing semantic categories and concepts, collectively known as the “semantic system” (Binder et al., 2009), and word representation (“semantic map”) has recently been mapped comprehensively with fMRI (Huth et al., 2016). Much of our understanding comes from the vast amount of neuroimaging and electrophysiological data recorded from both healthy subjects and neurogenerative disorder patients as well as the occasional invasive recording in patients during or in the work-up of surgery. However, despite this evidence, it is still not clear on what basis these representations differ.

One of the most prominent theories of semantic word processing is the grounded cognition or embodied cognition model. This model suggests that semantic knowledge resides in high-level perception and motor representation systems (Barsalou, 2008, 2010). In other words, a word is comprehended based on the modality-specific neural systems (for example, visual features such as color, shape and motor actions) that are associated with the definition of the word. Indeed, a number of fMRI studies have demonstrated that, for example, animate objects tend to cluster in the lateral part of the fusiform gyrus while inanimate or manmade objects cluster in the medial part (Martin and Chao, 2015). Furthermore, it has been shown that certain category mappings are based on specific shapes, such as “face” and “body” patches (Tsao et al., 2008). However, the embodied cognition framework has received considerable criticism, too (Mahon and Caramazza, 2008). For example, a number of lesion studies have been reported where damage to the brain's modal system led to category-specific deficits and to disproportionately preserved categories such as animals, foods, or artifacts (Barsalou et al., 2003; Caramazza and Mahon, 2003). An attempt to explain this along the lines of embodied cognition would need to resort to the most prevalent modality behind these categories, e.g. artifacts separated from animals and food as they call upon systems involved in manipulation or the action in response to experiencing the word, as evidenced by a larger activation in the left premotor cortex and the pre- and post-central gyrus (Hwang et al., 2009). However, category-specific deficits are hard to explain when relying only on embodied cognition, since many modality-specific systems might overlap between categories, and a single category might contain several distinct modality-specific features. This raises the question of how these modality-specific features are linked within one category and, conversely, when they span several categories, how they can be distinguished between categories. Increasing evidence suggests the existence of a modality-invariant integrative mechanism in addition to the modality-specific neural systems described in the classical model. This integrative component is called the semantic “hub,” and modality-specific neural systems are referred to as the “spokes” of the model. The hub and spoke model is regarded as the middle ground between the embodied and disembodied hypotheses (Mahon and Caramazza, 2008; Lambon Ralph, 2013).

However, the grounded cognition model, with its hub and spoke extension model (Lambon Ralph, 2013), can be criticized from several angles. First of all, modality-specific features of a concept are much less variant between different subjects when shown as a clear visual stimulus (image). However, when stimuli are conceptual in nature (such as words presented in written or spoken form), the perceptual and motor-sensory functions they refer to are much more difficult to control as the experience designated to the specific entities is to a large degree subject-dependent (Kemmerer, 2014). Secondly, which is particularly relevant to our study, the grounded cognition model only explains features that are rooted in the physical experience with the concept, limiting its applicability to concrete concepts. However, abstract concepts such as “democracy” or “emotions” are not explained by this theory, despite of their prominent presence in human language (Kemmerer, 2014).

The difference between processing abstract and concrete words has been addressed by a number of theories, the main ones being the dual coding and context availability theories (Kounios and Holcomb, 1994; Wang et al., 2010). The dual coding theory claims that two separate systems reside in the brain: a nonverbal “imagery” system that implements the modality-specific aspects of a concept (similar to the grounded cognition model), and a purely verbal “linguistic” system that is involved in the abstract form of language. According to the context availability theory, the processing of concrete and abstract words rarely occurs in isolation, but rather in the context in which they are understood. Since the context of concrete words is mainly constrained by the words' physical properties, understanding these words will again involve modality-specific brain systems as defined in the grounded cognition model. In the case of abstract concepts, however, context is more variable and experience-dependent.

Both theories put the grounded cognition model for different categories of concrete words into a larger framework that would also include abstract concepts. For example, some studies have attempted to define the “hub” of the previously mentioned hub and spoke model, and considered the anterior temporal lobes as a pan-modal hub for all concepts (so as to also include abstract concepts; Hoffman and Ralph, 2018). However, none of the aforementioned theories discuss the possibility of also having categories for abstract concepts and considers them homogeneous, despite the gamut of semantically significant subdivisions such as numbers, emotions, etc. The question remains whether an embodying ground can be defined for abstract concepts by means of well-defined domains. Despite some efforts in this regard (defining different abstract categories such as numbers and emotions; Kemmerer, 2014), we believe a good approach would be to search for a semantic space that can describe both abstract and concrete words. An important set of semantic attributes are the three dimensions according to the semantic differential scale proposed by Osgood and colleagues (Osgood et al., 1975). According to the study by Osgood, the affective meaning of linguistic terms can be quantified in three independent dimensions marked by the following polar adjectives: “evaluation” or “valence” (good- bad, pleasant-unpleasant, positive-negative), “potency” (strong-weak, heavy-strong, large-small), and “activity,” also called “arousal” in most current models (active-passive, fast-slow, sharp-dull) (Fontaine et al., 2013) thereby avoiding possible confusion with motor activity. These semantic attributes proved to be robust across age groups, cultures, and languages. Specifically, the neural correlates of valence and arousal for word processing have been investigated extensively in the past decade in the framework of emotion word processing (Kuchinke et al., 2005; Kissler et al., 2006; Lewis et al., 2007; Citron et al., 2013). For example, valence has already been suggested to ground abstract concepts in the same neural systems underlying basic emotions (Kousta et al., 2011; Vigliocco et al., 2014) since there seems to be a high level of interaction between abstractness and valence. In this study we extend this research by including the two other Osgood dimensions: arousal and potency. To the best of our knowledge, we are the first to propose these dimensions to dissociate their neural representation from that of abstract and concrete concepts. In order to unveil the networks activated by the Osgood dimensions, but also by abstract vs. concrete categories, and to detect any differences in the evoked spatio-temporal activity patterns, we need tools that match the temporal resolution of the underlying neural mechanisms. The temporal course of word processing is crucial for the functional interpretation of brain activity, e.g., to determine during which process we can see the effect of lexical or semantic properties.

A crucial limitation of all the aforementioned theories is that they have predominantly relied on functional neuroimaging techniques such as fMRI and PET, which are limited in temporal resolution (Bookheimer, 2002). As word recognition involves stages that unfold on a scale of milliseconds, such as visual encoding, lexical activation, and semantic presentation (Laszlo and Federmeier, 2014), the temporal resolution should be on par with the dynamics of these processes. Whether these stages are sequential or partially parallel and interactive is still debated (Hauk et al., 2006; Grainger and Holcomb, 2009), however it is clear that hemodynamic imaging cannot track the activity progression or resolve differences in activity patterns between words (Helenius et al., 1998; Bentin et al., 1999). On the other hand, studies that involve lesions and electrical stimulations do not provide the proper scope for the large-scale networks that are possibly involved.

By virtue of its excellent temporal resolution, the EEG technique has been hailed for gauging the brain's semantic processes. Studies using EEG/ERP recordings have successfully distinguished differences in word categories in different stages of semantic processing, starting from detecting differences in word length and word frequency around 100 and 200 ms (Hauk et al., 2006) to the distinction in processing semantic categories, such as abstract vs. concrete concepts (Bentin et al., 1999), animals vs. tools (Simanova et al., 2010) and more generally natural objects vs. artifacts (Kiefer, 2001), around 400–500 ms (N400 potential).

The main drawback of traditional scalp EEG is its relatively low spatial resolution, which falls short in detecting differences in cortical network activation. For example, different aspects of semantic activity and language comprehension could be coded by different neural generators during the same time period, but go unnoticed when inspecting the N400 scalp distribution (Nobre and McCarthy, 1995; Lau et al., 2008). It is clear that spatial resolution is equally important for obtaining a complete picture of the neural activations underlying the semantic processes. However, by using high density EEG and source localization, one can reconcile the temporal and spatial resolution requirements needed to uncover the spatio-temporal dynamics of neural activity with an accuracy in the range of millimeters and milliseconds. We have adopted this technique to investigate the contribution of lexico-semantic features of word processing (orthographic/phonological, lexical access and semantic processing) and their spatio-temporal distribution in the brain. We hypothesize that, by performing statistical analysis directly on the sources as opposed to scalp-level analysis, fine-grained differences previously obscured in EEG studies due to volume conduction can be revealed. In order to identify patterns of cortical activation we caution against scalp-level analysis, since many effects might be overlooked, as shown in this paper. To evaluate our paradigm, which calls upon the said semantic features, we developed a mass-univariate analysis approach on source level to discriminate between the different patterns of cortical activation.

## Materials and Methods

### Participants

Twenty-two paid, healthy, native Dutch-speaking volunteers (9 males, 3 left-handed, average age 22 ± 4 years) participated in the study. The study was approved by the UZ Leuven ethics committee and conducted according to the latest version of the Declaration of Helsinki. All recruited subjects were first informed about the goal of the experiment, what would be expected from them, and what would be done with the collected data (privacy), after which they were invited to read and sign the informed consent form. No participant reported any history of neurological or psychiatric disorders. All participants had normal or corrected to normal vision.

### Word Stimuli

We used 159 Dutch nouns combined from various categories: emotions and other abstract concepts, shapes, tools, colors, vegetables, food, fish, insects, furniture, sport disciplines, birds, and mammals.

We use the following semantic dimensions to discern our categories: word abstractness, potency, valence, and arousal. For our analysis all nouns were grouped based on these dimensions (note that within a single trial during the experiment, words could belong to any of the aforementioned semantic dimensions), and these dimensions were based on the three Osgood ratings (Moors et al., 2013) as well as on the concreteness ratings of Dutch words (Brysbaert et al., 2014). The words were chosen to have a score below 3.5 or above 4.5 for both poles of each dimension of the Osgood ratings, so as to ensure a statistically significant difference between the two groups on the same dimension (e.g., weak vs. strong for potency). The same principle applied to the abstractness ratings, abstract words should have a concreteness score below 2.5 and concrete words above 3.5. A *t*-test analysis showed a statistically significant difference between the two groups of each semantic dimension and the abstract and concrete groups based on their concreteness rating (*p* < 0.05, high valence: *m* = 4.98, std = 0.50, low valence: *m* = 2.66, std = 0.52, high arousal: *m* = 5.13, std = 0.40, low arousal: *m* = 3.18, std = 0.23, high potency: *m* = 5.07, std = 0.37, low potency: *m* = 3.10, std = 0.24, abstract: *m* = 1.89, std = 0.43, concrete: *m* = 4.77, std = 0.28). These semantic categories were then further controlled along each dimension for word frequency, orthographic neighborhood size and word length (i.e., number of letters in a word) ratings taken from the Dutch CLEARPOND software (Marian et al., 2012): repeated measure ANOVA showed no significant difference between dimensions for word frequency and orthographic neighborhood size (for all dimensions, *p* > 0.05). In the case of number of letters, no significant difference was between dimension potency and valence (*p* > 0.05), while differences in word length between abstract and concrete words and high and low arousal words were significant (*p* = 0.0043 for abstractness and *p* = 0.0027 for arousal). For these two word dimensions, for which we were not able to entirely control for word length, we repeated the same analysis by also including word length as a random factor. This analysis did not result in an elimination of any of the previous outcomes; however, in one case, these dimensions yielded additional activity regions (cf. discussion section). The entire dataset can be found in the Supplementary Material in Appendix A.

### Experimental Paradigm and Set-Up

Participants were tested in a sound-attenuated and darkened room with a constant temperature of 20 degrees Celsius, sitting in front of an LCD screen at a distance of about 70 cm. EEG data was recorded using 128 active Ag/AgCl electrodes (SynampsRT, Compumedics, France) according to the international 10-5 system. Two of these electrodes served as ground (AFz) and reference (FCz). The EEG signal was recorded at a 2 KHz sampling rate and down sampled to 500 Hz. All electrodes were mounted in an electrode cap, placed on the subject's head, for which we established the position of electrode Cz as the central point between inion, nasion, and the two pre-auricular points (Easycap, Germany). The cap was later used to obtain the electrode positions as pre-specified in the Brainstorm toolbox for source reconstruction. Conductive gel was applied in each of the electrode holes to ensure contact with the scalp.

During the experiment, in each trial, four white words on a black background were shown consecutively for 500 ms each, separated by a black screen for 1.2 s (inter-stimulus interval) with a jitter of ±200 ms. Three of these words were members of the same semantic category and the remaining word (called “filler”) could either be a member of the same or a different semantic category. The order of this filler in the 4-word trial was randomized. Examples of such trials are shown in Table 1. We will refer to the remaining 3 words in the trial as “target words” since they will be analyzed and the filler word discarded.

**Table 1:** Example trials.

**Trial type**	**word 1**	**word 2**	**word 3**	**word 4**
*homogeneous trial*	ansjovis (anchovy)	kabeljauw (codfish)	inktvis (squid)	***snoek (pike)***
	knoflook (garlic)	ui (onion)	***asperge (asparagus)***	selderij (celery)
	tang (pliers)	zaag (saw)	bijl (ax)	***schop (shovel)***
	***libel (dragonfly)***	vlieg (fly)	mier (ant)	wesp (wasp)
non-homogeneous trial	adelaar (eagle)	kanarie (canary)	specht (woodpecker)	***kokosnoot (coconut)***
	zilver (silver)	***brand (fire)***	groen (green)	bruin (brown)
	***piano (piano)***	konijn (rabbit)	kalkoen (turkey)	tijger (tiger)
	jaloezie (jealousy)	vreugde (joy)	afkeer (aversion)	***acteur (actor)***

*During the experiment all words were presented in Dutch. The fillers are presented in bold-italic, with related fillers in the homogeneous trials and unrelated fillers shown in the non-homogeneous trials. The three remaining words are the target words. English translations (between brackets) are for illustrative purposes only*.

Each trial started with a fixation cross that would cue the subject to gaze at the middle of the screen. After that, words were displayed and a cue was shown to prompt the subject to press the left mouse button if they thought all four words came from the same category (homogeneous trials) or the right button if otherwise (non-homogeneous trials). This semantic category matching task was chosen in order to ensure the proper depth of word-processing in our subjects (semantic processing, instead of mere lexical access, as would be expected for a lexical decision task). Note that the identification of these categories was not the goal of our study but rather used as a task for the subject. Subjects were asked not to click the button before the cue appeared in order to prevent contamination of our ERPs-of-interest with motor-related ones. After pressing a mouse button, they received visual feedback, which did not reflect the correctness of the trial but rather served as a reminder for the function of each mouse button (“goed!” (correct!) for the left button press, and “fout!” (wrong!) for the right button press).

The order of the trials was pseudo-randomized in a way that the same semantic category would not appear in two consecutive trials. Every subject repeated the experiment twice, with a break of about 20 min between the two sessions during which they performed another experiment so as to mitigate repetition effects. In total, the experiment lasted 30–40 min. The stimuli were presented using Matlab's Psychophysics Toolbox (Brainard, 1997).

### EEG Signal Pre-processing

EEG data was re-referenced offline from the original ground and reference electrodes to a linked mastoid one and filtered using a 4th order Butterworth filter in the range of 0.1–30 Hz. Even though the choice of the reference does not affect the inverse localization of neural active sources (Geselowitz, 1998; Pascual-marqui, 2007), we chose the linked mastoid reference because it was shown to yield a clearer N400 scalp response, which is one of the main ERP components used in the study of semantics (Marí-beffa et al., 2007; Kutas and Federmeier, 2011). For this reason, the linked-mastoid reference is the most commonly used one in linguistic ERP studies (Marí-beffa et al., 2007). The data was epoched using a window starting 100 ms prior to the presentation of the stimulus of interest (each of the 3 target words) until 1,000 ms post-onset. The average amplitude of 100 ms pre-stimulus signal was used to remove the post-stimulus EEG signal baseline. Bad channels were eliminated for each subject based on a visual inspection of the data (an average of 18 channels were eliminated per subject), After this, trials in which the EEG amplitude on any of the electrodes exceeded ±150 μV were excluded as they could be due to motion artifacts.

### Source Localization

For our source reconstruction analysis we used the Brainstorm toolbox (Tadel et al., 2011), freely available under the GNU general public license. The default anatomy was based on the ICBM-152 template. For the forward model we used OpenMEEG BEM (Gramfort et al., 2010), in which case the cortex was divided into 15,000 dipoles. The noise covariance matrix was obtained by merging the matrices calculated from the baseline of all selected trials. As our inverse modeling method, we used minimum norm estimates to estimate the sources and sLORETA (Pascual-Marqui, 2002) to normalize the estimated source density, as it has shown to yield zero localization errors in the absence of noise and to support the reconstruction of multiple sources. The source density is normalized at each point by a function of the data covariance and is unitless. Source orientation was constrained to be orthogonal to the cortical surface. The signal-to-noise ratio (SNR) was set to the default value suggested by the Brainstorm Toolbox (SNR = 3). In addition, sulci are not taken into consideration during our analysis, as accurate source localization in these regions is implausible, as stipulated in Brainstorm's documentation.

In order to verify the correctness of our procedure, we attempted to reproduce the results using different source localization algorithms, as is recommended by Mahjoory et al. (2017). We did not pursue the entire statistical procedure; however, we did analyze our initial results by taking the average over all trials regardless of semantic features, using the four methods available in the brainstorm toolbox: wMNE, dSPM, sLORETA, and unconstrained sLORETA. Manual inspection of the results with these methods revealed that the spatial distributions are similar over time albeit with different degrees of spatial smoothing.

### Statistical Analysis

For both scalp and source domain data, a mass-univariate approach using a linear mixed effect model was adopted (Verbeke and Molenberghs, 2000) with subjects and word length taken as random effect and semantic feature labels as fixed effects. In the case of the Osgood dimensions, we also corrected for the concreteness of the word by including it as a random effect. The dependent variable in the scalp domain was EEG amplitude and in the source domain it was dipole amplitude. Both averaged over 50 ms time bins between 0 and 900 ms. The final model can be seen in Equation 1:

(1) $$\begin{array}{l} ‘averagedamplitude^{\prime }\sim semanticfeature+\left(1|Concreteness\right)\\ +\left(1|Subjects\right)+\left(1|WordLength\right), \end{array}$$

for the Osgood dimensions, and

(2) $$\begin{array}{l} ‘averagedamplitude^{\prime }\sim Concreteness+\left(1|Subjects\right)\\ +\left(1|WordLength\right) \end{array}$$

for abstract vs. concrete words. In the source domain, averaging was performed for each of the 15,000 dipoles. Test results with *p*-values below 0.05 were considered significant. In cases such as ours, where dipoles are not independent from each other, and the number of dipoles is extremely large, Bonferroni- or FDR corrections are not appropriate as they will eliminate true positives (Cohen, 2014). Instead, we corrected for multiple comparisons using cluster-based inference adapted from the random field theory (Friston et al., 1994); as a threshold we took 3 cm^2^ of cortical surface as the minimal cluster size. The reasoning behind this threshold was based on a simulation study of the localization accuracy and is explained below. The same principle was applied for correcting over time, e.g., we only took those regions into consideration that were statistically significant for at least two consecutive time bins. When the measurement window and electrode sites are not known a priori, as in our case, one could rely on permutation tests to solve the multiple comparison problem in time and space simultaneously (Luck and Gaspelin, 2017). However, we felt the linear mixed effect to be a more appropriate model since it can account not only for inter-subject variability but also variability of any additional parameters (such as word length) and, therefore, has more power to detect small differences. Most importantly, the model allows us to control for the concreteness of the word when evaluating the Osgood dimensions, which is an important feature for our claim to suggest the dimensions as a way to ground both concrete and abstract words in the same underlying neural systems. Additionally, as suggested in many studies (Feise, 2002), we looked at effect size, specifically Cohen's d-effect size. Note that, as is the case with EEG studies, especially single word comprehension studies, differences are likely to be small and effect sizes of 0.01~0.1 are to be expected (Kutas, 1993). In the scalp domain we also used cluster-based inference over electrodes by taking a cluster size of 10 adjacent electrodes, and only accepted clusters that were significant for at least 2 consecutive time bins, which would mean effects that last about 100 ms, which corresponds to the approximate duration of transient EEG events (Koenig et al., 2002). It is very important to note that, since we performed the statistical analysis on relative values of dipole amplitudes (we do not rectify the signal for the mixed effect model), our results measured the distance between two conditions, showing the correct polarity of the signal. This is the case since the sign of the dipole amplitudes depends on the orientation of the source. However, the sign is ambiguous and cannot be used to claim that the brain response is stronger in one condition compared to another. Once our results are acquired, when studying a particular region of interest, rectified signals can be used to know which condition generates a larger response. This is possible since ||A|-|B||≤|A-B|, which implies that the effect of rectified signals will always be smaller, therefore there is no need to perform statistical analysis on the rectified signals separately. Further information can be found in the tutorials on difference estimation provided by Brainstorm (http://neuroimage.usc.edu/brainstorm/Tutorials/Difference). In addition to the aforementioned, we did not take into account regions that were located on the bottom of the sulci or in the interhemispheric fissure (we did take into account the wall of the gyri, but eliminated results at the bottom of the sulci as defined by Brainstorm), as source localization in these regions is suggested to be implausible and difficult to detect from EEG-based source localization.

**Figure 1:**
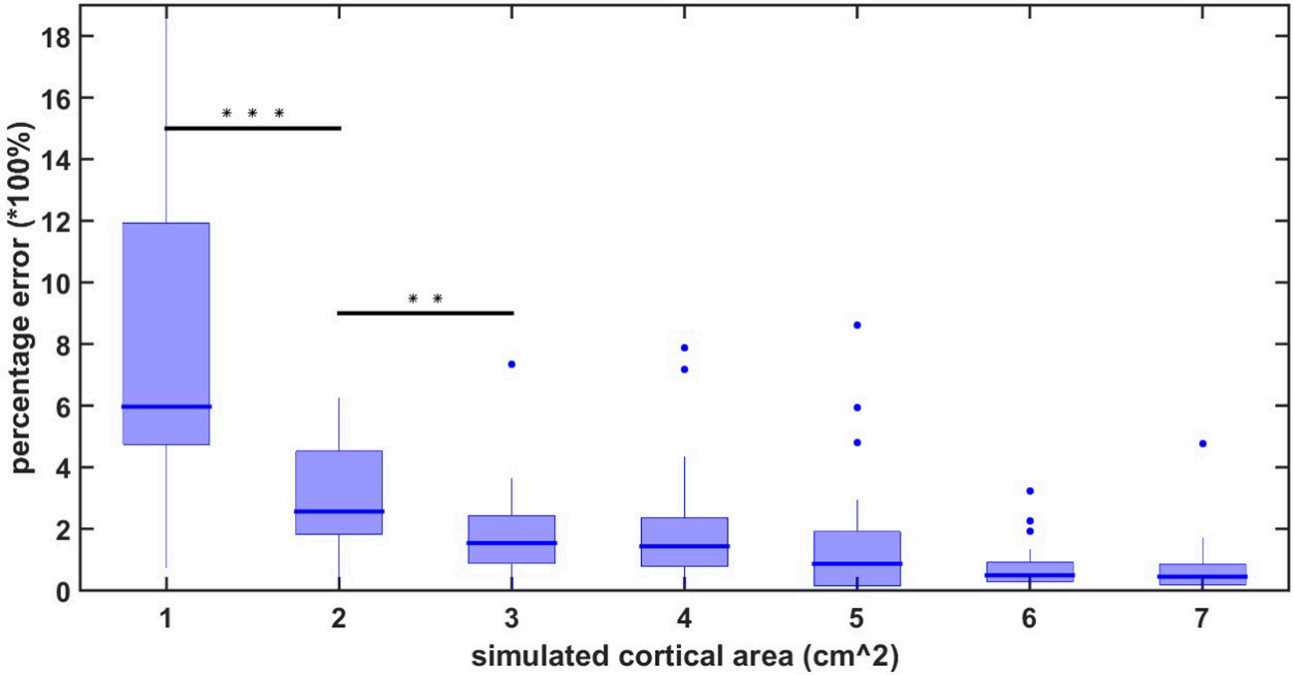
localization percentage error for simulated source activity.

The average localization error using the high-density EEG method applied in this study has previously been reported to be 10.5 mm for realistically shaped head models (Cuffin et al., 2001). However, these results are still under dispute (Song et al., 2015). Therefore, to ensure that the results of our statistical test are not merely localization inaccuracies, we performed a simulation study to measure the amount of crosstalk between source-localized areas as a function of the spreading of activation. We simulated a signal consisting of a linear combination of sinusoids with frequencies ranging from 10 to 100 Hz. We applied this signal to randomly chosen regions on the cortical surface with different square sizes. We chose 20 random areas for each simulated size of 1, 2, up to 7 cm^2^ of cortical surface. For each of these simulated regions, we applied the forward model to obtain simulated EEG signals on the scalp, and then applied the inverse solution to obtain an estimate of the spread of activity on the cortical surface. Since the activation of methods that employ L2 regularization, such as sLORETA's, will exhibit nonzero activity at all-time points and spatial locations (Gramfort et al., 2012), we defined the spreading of activation as the area outside which the amplitude of the dipoles falls below 50% of the maximum amplitude. The area of activity after determining the forward and inverse solutions was compared with the initially simulated activity in order to obtain a measure of the crosstalk (or spatial accuracy of the model). Figure 1 shows the percentage error (calculated as $\frac{abs\left(calculated\;cortical\;area-simulated\;cortical\;area\right)}{simulated\;cortical\;area}$) between the initially simulated and calculated area for surface region sizes between 1 and 7 cm^2^. As one can observe, the statistical difference between the region sizes drops below significance after 3 cm^2^ of simulated activity. Our cross-talk simulations confirmed that, for cortical surface activity above 3 cm^2^, the extent of spatial spreading does not differ significantly between any two cortical areas larger than 3 cm^2^. Therefore, in our linear mixed effect model on the cortical surface, we only retained areas of significance that where larger than 3 cm^2^.

## Results

### Scalp Analysis

**Figure 2:**
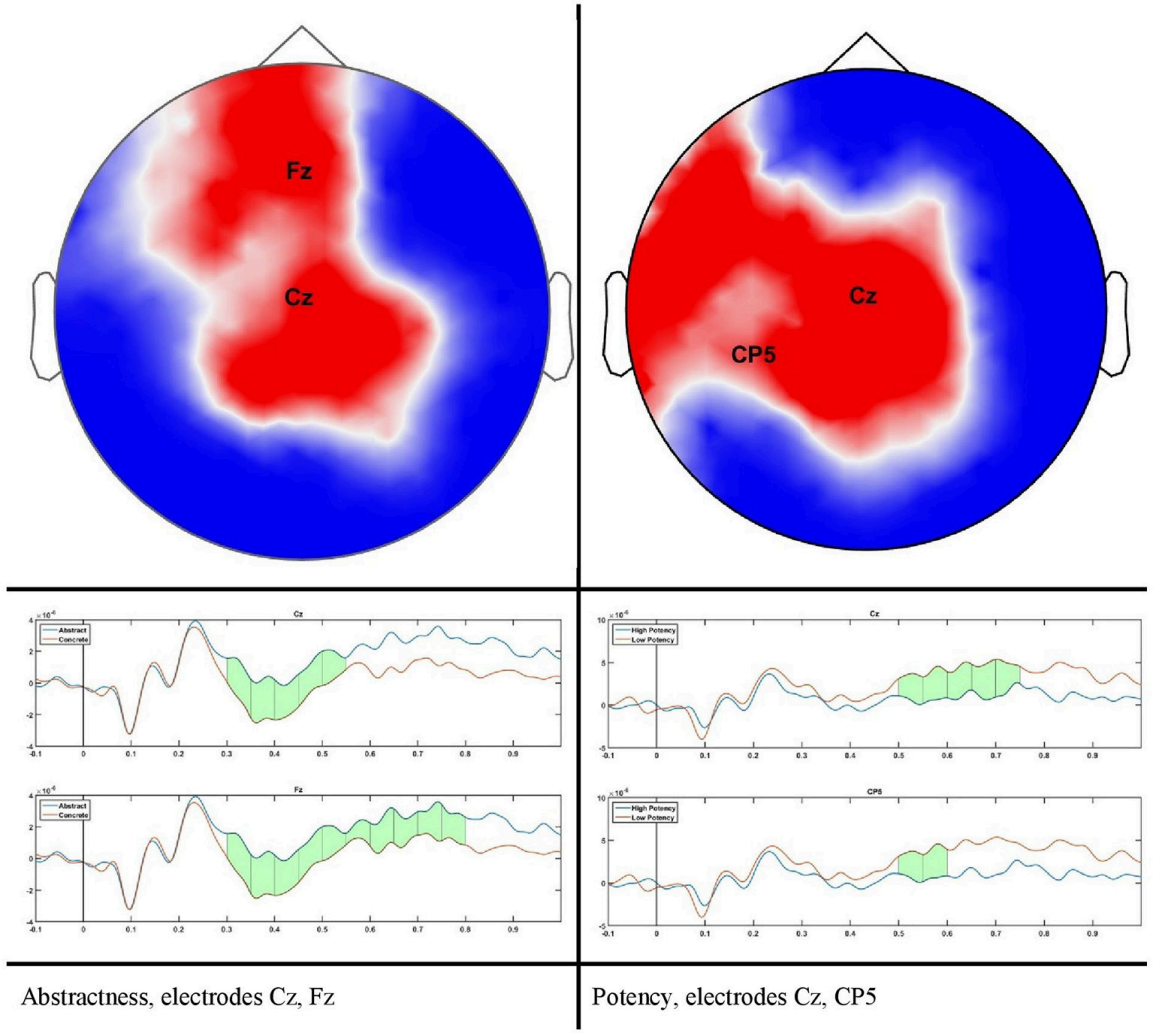
scalp plot for significant differences and time course for two sample electrodes as indicated in scalp plots.

**Figure 3:**
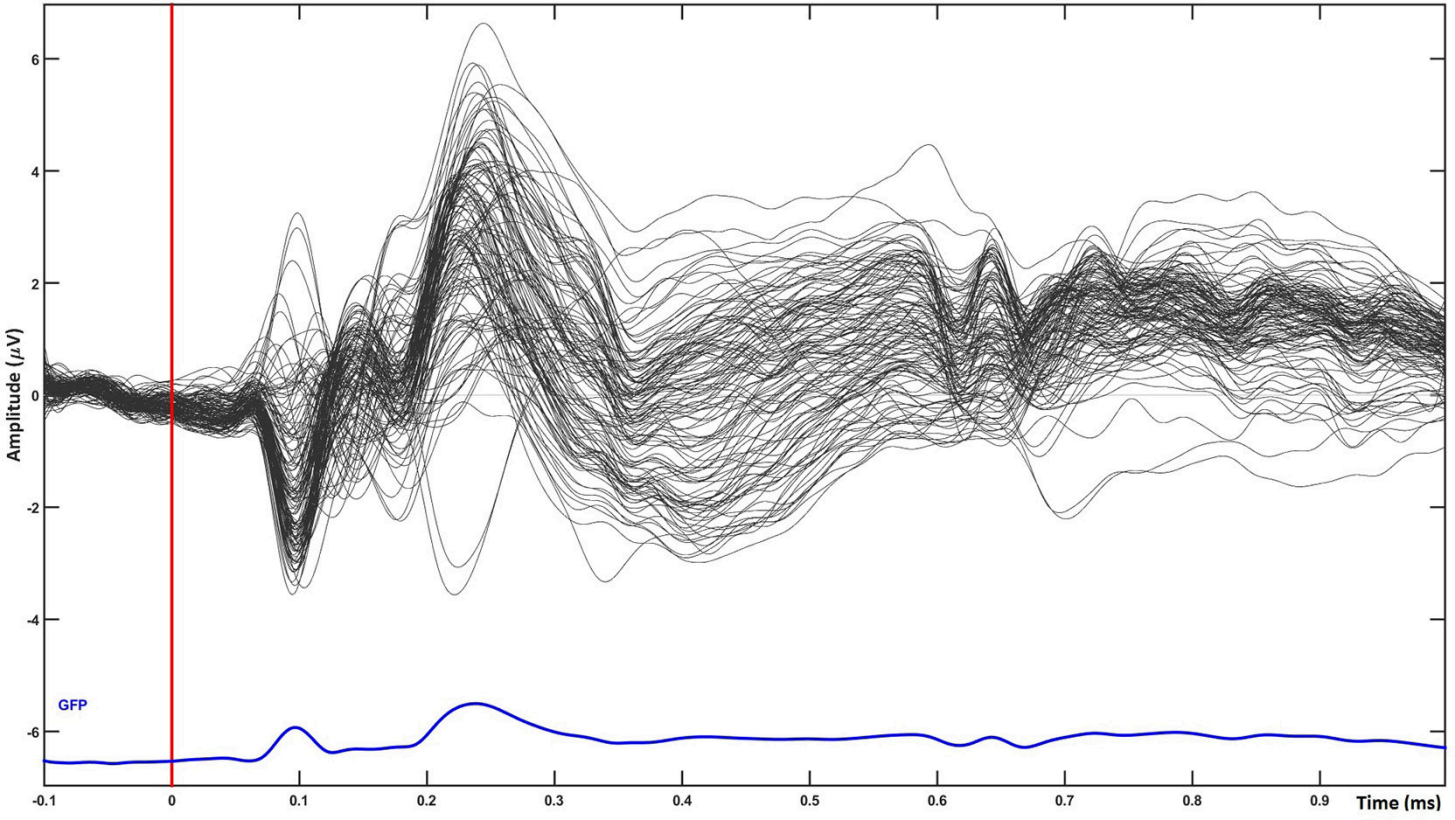
temporal plot for single word processing averaged over all groups of words (grand average of all trials). The plot shows the quality of ERP data used for source analysis.

The scalp results obtained from the statistical analysis described previously are shown in Figure 2. A grand average of all trials shown in Figure 3 confirms the quality of our ERP data.

For abstractness, significant differences can be seen during the time course of 300–500 ms on a cluster of 23–28 channels (*P* = 0.0106, Cohen's *d* = 0.042), and for potency during 500–700 with a cluster size of 21–24 electrodes (*P* = 0.0383, Cohen's *d* = 0.0642). No results remained after correcting for multiple comparisons for the dimensions of arousal and valence. This does not mean that no differences could be found on scalp level, but rather that our threshold for the cluster size is conservative.

### Source Analysis

In order to evaluate any theory on word processing, a spatio-temporal view of brain activity is needed. When a single word is processed, categorized, and stored in semantic memory, a distributed network of cortical activity will emerge, starting to spread from the occipital areas toward the anterior temporal lobe (Borghesani, 2017). The entire activation network has been proposed to involve the left temporoparietal, angular, inferior and middle temporal gyri. When subjects are required to relate word meanings then several regions of the frontal lobe are additionally activated (Silva-pereyra et al., 2003).

**Figure 4:**
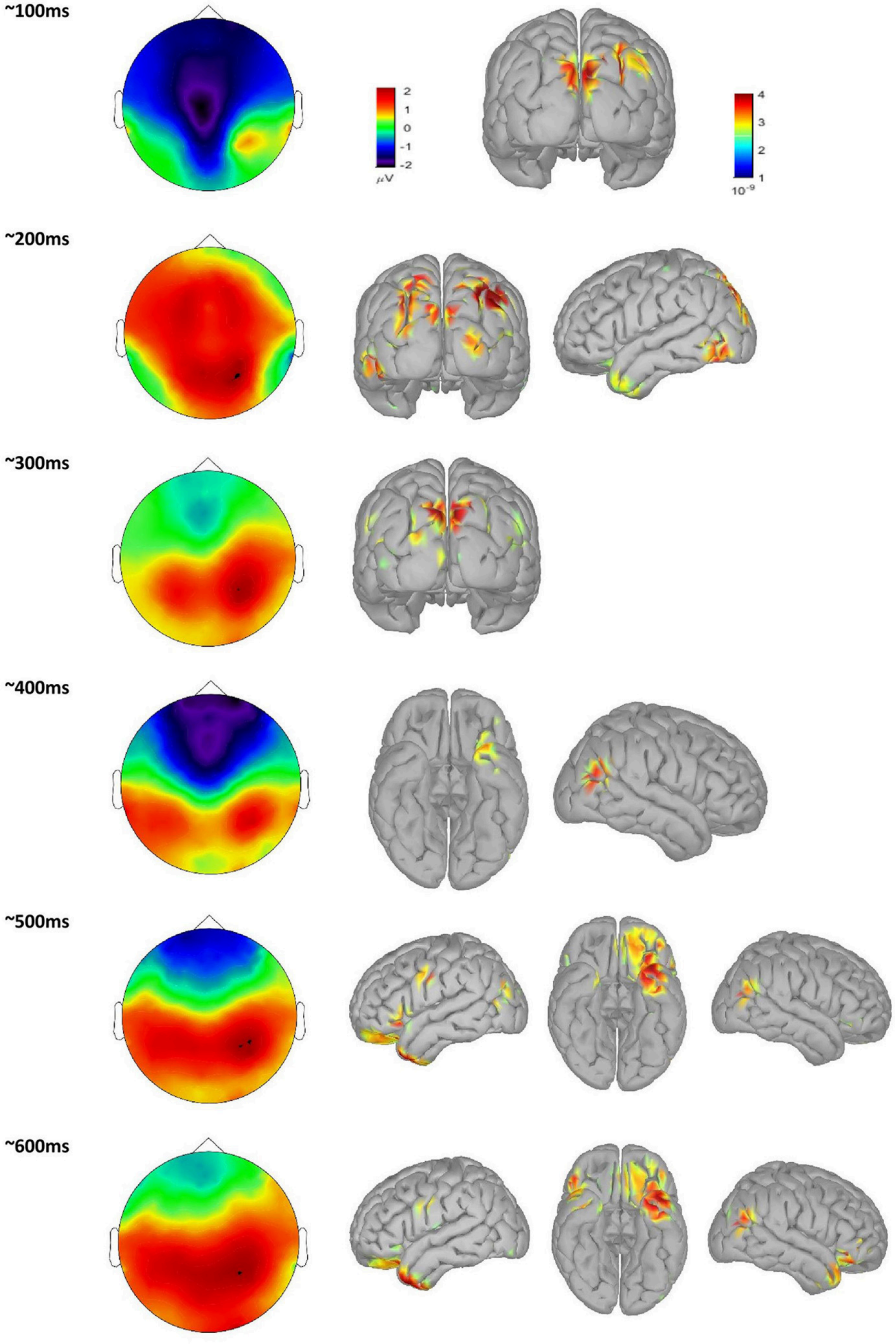
scalp plot of time course of single word processing averaged over all groups, with associated source localization maps.

When averaging across all word groups, source-localized brain activity can be seen (Figure 4) at different stages of lexico-semantic processing. The early stages start with visual processing in the occipital lobe, then areas are recruited along the ventral stream, specifically the posterior occipital area of the left hemisphere around 200 ms, until the posterior temporal lobe is reached in the time window of semantic processing (400 ms), and onwards anterior temporal, inferior frontal and orbital gyrus are included at later stages. This stream of activation has been previously shown with source localization studies (Borghesani, 2017). Therefore, together with the fact that our trials have only restricted variability in terms of noise as can be seen in Figure 3, this confirms the quality of source localization entering our statistical analysis.

### Results of Statistical Analysis on Source Data

#### Abstract vs. Concrete

**Figure 5:**
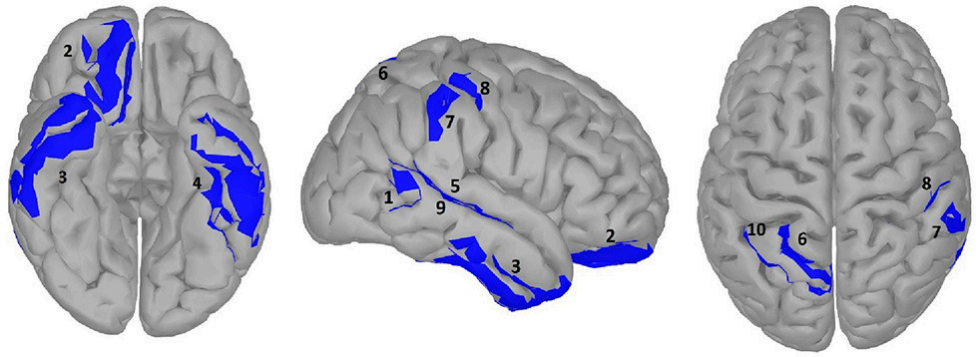
significance map for abstractness.

#### Arousal

**Figure 6:**
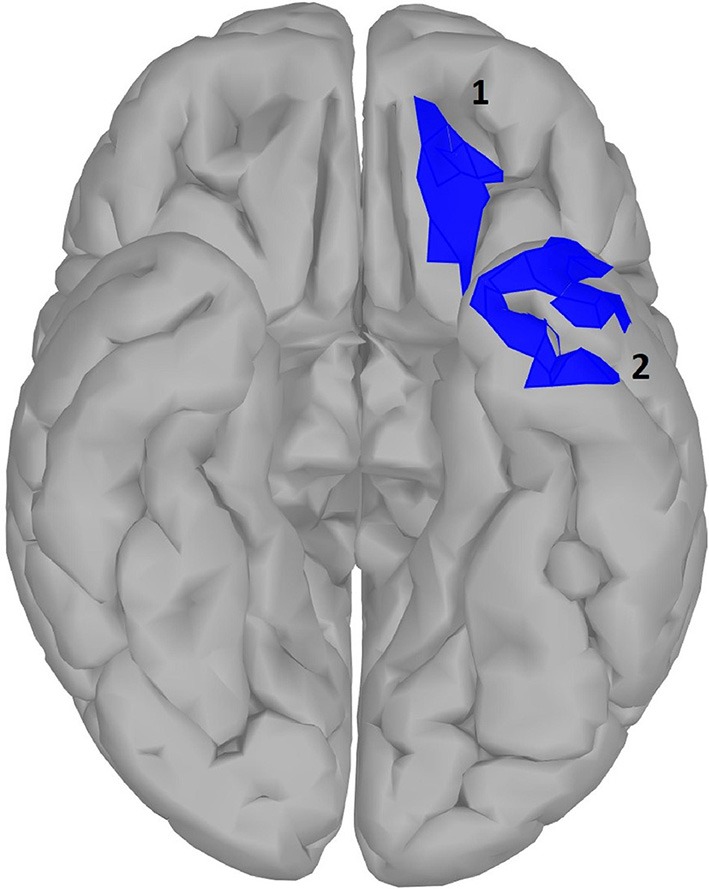
significance map for arousal.

#### Potency

**Figure 7:**
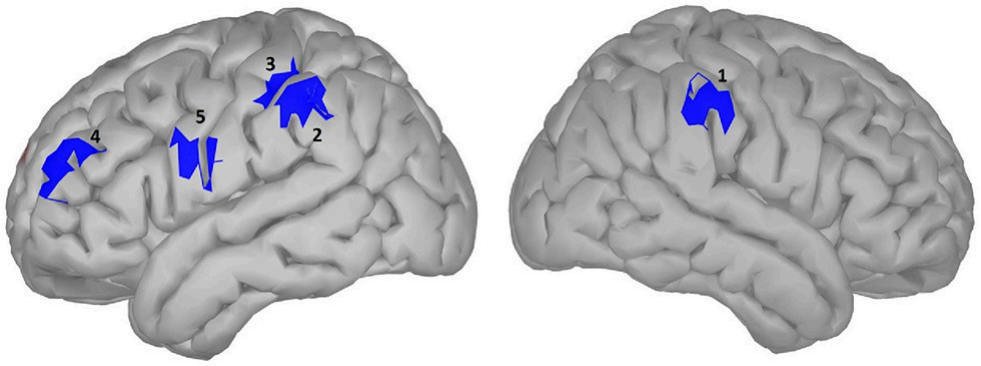
significance map for potency.

#### Valence

**Figure 8:**
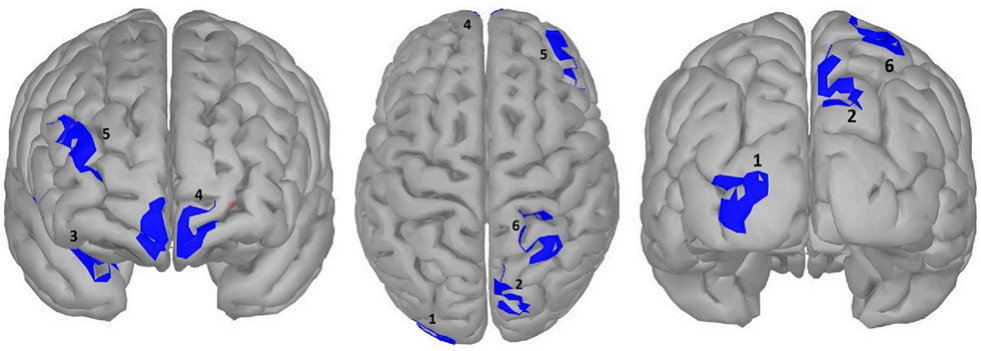
significance map for valence.

## Discussion

### Effect of Abstractness: Abstract vs. Concrete Words

A large difference was observed in the scalp domain during the time window of semantic processing, located centro-parietally (Figure 2, 300–500 ms), in agreement with previous reports on the effect of abstractness in word processing (West and Holcomb, 2000; Kanske and Kotz, 2007). This result translated to a few regions in the source analysis (Figure 5: regions 2 and 3, and Table 2). Significant differences in sources between abstract and concrete words are present very early on in the posterior parietal areas (the time-interval for visual and orthographic processing) followed by the left and right temporal cortex and the right inferior frontal cortex (time-interval of semantic processing), and the occipito-parietal areas toward the end of the 900 ms time-window. Regions located in the left hemisphere such as the fusiform and middle temporal gyrus are akin to those found with fMRI studies (Wang et al., 2013). Additionally, a large involvement of the right temporal cortex in processing abstract words, especially during the time interval of semantic retrieval, suggests the involvement of more brain areas in the processing of abstract concepts in the right hemisphere. This is similar to the work of Kiehl et al. (1999), who suggested a right hemisphere neural pathway for processing abstract words. For most brain areas that were, according to Perani et al. (1999), involved in processing abstractness, we observed larger activity in response to abstract compared to concrete words. However, unlike their study, where activity was observed only in the left posterior temporal and bilateral frontal cortices, we observed additional activity in the right temporal cortex and the occipito-parietal cortices in the right hemisphere, consistent with some PET studies suggesting an interpretative role of the right hemisphere in processing abstract language (Beauregard et al., 1997; D'Esposito et al., 1997). A possible reason for why our study revealed more areas in the right hemisphere, compared to similar fMRI studies such as Kiehl's (Kiehl et al., 1999), could be that with EEG, given its excellent temporal resolution, short-lasting activations are more visible than with fMRI.

**Table 2:** Statistical results for significance map of Figure 5 (abstractness).

**Region**	**Time window (ms)**	**P value (corrected)**	**Polarity**	**Stronger condition**	**Cohen^**′**^s d**	**Size (cm^**2**^)**	**Estimate (10^**−10**^)**	**Confidence interval (10**^****−10****^ **)**	
1 right posterior MTG	100–200	0.013	A>C	|A|>|C|	0.040	4.42	5.31	[2.86	23.70]
2 right medial orbital gyrus	450–650	0.020	A>C	|A|<|C|	0.025	22.96	10.80	[3.18	39.17]
3 right anterior inferior temporal gyrus and MTG	350–650	0.023	A>C	|A|>|C|	0.036	24.18	15.86	[4.63	66.84]
4 center anterior MTG and ITG	300–550	0.029	A>C	|A|>|C|	0.032	37.04	14.96	[1.02	59.71]
5 center STG/STS^*^	250–700	0.015	A>C	|A|>|C|	0.041	12.71	7.72	[4.68	34.95]
6 center superior parietal gyrus	700–1,000	0.018	A>C	|A|<|C|	0.038	10.25	6.74	[2.89	29.33]
7 right inferior parietal gyrus	0–100	0.018	A < C	|A|<|C|	0.040	4.68	2.87	[−12.77	−1.50]
8 right postcentral gyrus	0–100	0.010	A<C	|A|>|C|	0.045	9.01	2.33	[−11.16	−2.00]
9 center MTG/MTS^*^	300–700	0.018	A<C	|A|>|C|	0.039	14.17	9.33	[−40.83	−4.22]
10 center middle parietal gyrus	700–1,000	0.019	A #x0003C;C	|A|<|C|	0.036	9.91	7.47	[−31.53	−2.22]

*A, Abstract; C: Concrete*.

* *The activation is located on the wall of sulcus but does not include the bottom*.

Another important fact to mention is the time window of semantic processing, usually linked to a scalp ERP component referred to as the N400, a negative amplitude deflection that peaks around 300–500 ms in response to a potentially meaningful stimulus (Lau et al., 2008; Kutas and Federmeier, 2011). Most studies observe a larger N400 amplitude for concrete words than for abstract ones (Kounios and Holcomb, 1994), as is also the case with our study (Figure 2). However, there is no consensus on the location of this difference as some suggest it to be in the right hemisphere (Kounios and Holcomb, 1994) while others suggest the opposite (Kiehl et al., 1999). Kiehl argues that this apparent contradiction could be mainly due to a different choice in electrode referencing. We suggest to take this one step further by arguing that, in addition to the choice of the electrode reference, the lateral direction of scalp activity is not always an indication of the underlying source as, depending on the orientation of dipole activity, scalp activity can originate from a region that is not directly beneath it (Gloor, 1985). However, this issue can be resolved when using source reconstruction as the location of the neural sources is reference-independent (Michel et al., 2004). Therefore, we claim our results to be more objective than those of previous scalp ERP studies and, in this way, we can conclude that the right hemisphere plays a bigger role in the difference between brain activation in response to abstract and concrete words.

### Effect of Arousal: Active vs. Passive Words

Arousal and valence have been suggested to be part of a two-dimensional model of emotional word processing (Citron, 2012; Citron et al., 2013). In this framework, some studies have questioned whether these dimensions are actually distinct or correlated (Bradley and Lang, 1999). However, in our study no correlation was found between the Osgood ratings of arousal and valence in our word dataset. In the temporal domain, we did not observe any difference large enough to outlive correction between high and low arousal words. Similar EEG studies, using a lexical decision task to investigate the effect of arousal in single word processing, did observe ERPs in early time windows to be modulated by arousal (Hofmann et al., 2009; Conrad et al., 2014) but in these studies stimuli are controlled for valence (words were all negative), which was not the case with our study. Additionally in these studies, window selection was less conservative compared to ours. When using alternative modalities such as images of brand logos instead of written stimuli, again no effect of arousal was observed (Schaefer and Rotte, 2010). Other studies (Lewis et al., 2007; Aryani et al., 2018) observed effects of increasing arousal in deep structures such as the amygdala and putamen, which is very difficult to observe with EEG (see discussion). However, in our source localization analysis (results shown in Figure 6 and Table 3), we did observe two localized areas in the left hemisphere similar to those seen by Aryani et al. (2018), temporal pole and medial orbital gyrus, that were modulated by word arousal. These areas resemble the left superior and middle temporal gyrus and the middle frontal gyrus reported by Kawachi et al. (2011), which was closest to ours although it was done with fMRI. Differences for both these regions occur relatively late in word processing, around 650–800 ms. The late effect can be explained by the claim that during the integration process of a word in working memory (600–800 ms), words with high arousal require more activation from the brain area involved in word integration (Lau et al., 2008), which can be seen only in the medial orbital gyrus. On the other hand, the left temporal pole, including the anterior superior temporal gyrus, shows increased activity for passive words. Note that in this study, the parietal gyrus was also mentioned to exhibit more activity, a plausible result since the superior parietal cortex is suggested to be involved in attention allocation (Ptak, 2012; Gonzalez et al., 2015). Given this, we can assume that for the initial processing of active words less attention is needed and therefore their retrieval easier. It is interesting to mention that when we took word length as an additional random variable into account in our model, the superior parietal cortex indeed showed significantly higher activation for passive words in the early time windows (100–200 ms, results not shown).

**Table 3:** Statistical results for significance map of Figure 6 (arousal).

**Region**	**Time window (ms)**	***P*-value (corrected)**	**Polarity**	**Stronger condition**	**Cohen^**′**^s d**	**Size (cm^**2**^)**	**Estimate (10^**−10**^)**	**Confidence interval (10**^****−10****^ **)**	
1: left medial orbital gyrus	650–800	0.019	HA>LA	|HA|>|LA|	0.040	6.65	11.07	[4.82	48.26]
2: left temporal pole	600–800	0.019	HA>LA	|HA|<|LA|	0.037	13.60	15.31	[3.57	63.31]

HA, High Arousal; LA, Low Arousal.

### Effect of Potency: Strong vs. Weak Words

We did not observe any effect of potency during the time window of semantic processing in either the scalp or the source domain, but in a later window an effect was observed, mainly a smaller amplitude for high potency stimuli compared to low ones that were lateralized in the left hemisphere, specifically the inferior parietal and the superior frontal and inferior precentral gyrus, where only the inferior parietal lobe showed higher activation for stimuli with a high potency value. The inferior parietal region was mirrored in the right hemisphere, albeit in an earlier time-window than the left hemisphere, since it was activated in response to word potency during lexical access and semantic retrieval, while other areas were activated starting from a late time window (550 ms) and lasted until the late stages of word processing (around 800 ms), (scalp domain results shown in Figure 2, source domain in Figure 7 and Table 4). The larger activation for low compared to high potency in the superior frontal cortex has been reported in several other studies, using both fMRI and EEG (Skrandies, 1998; Schaefer and Rotte, 2010; Kawachi et al., 2011). This could be because high potency words are processed easier (lexical access and semantic retrieval), as evidenced by physiological responses recorded during behavioral tests (Bradley et al., 2001), reducing the load of working memory and cognitive control and the involvement of the pre-frontal cortex (Macdonald et al., 2000). Considering these studies involve both written and visual stimuli, we believe this activation to be independent of modality. Studies involving this dimension have been generally scarce, since the majority of studies investigating the neural mechanism of emotional processing focus on Osgood's two other affective meaning dimensions, valence and activity (Fontaine et al., 2013). However, as our study shows, the potency dimension should be overlooked.

**Table 4:** Statistical results for significance map of Figure 7 (potency).

**Region**	**Time window (ms)**	***P*-value (corrected)**	**Polarity**	**Stronger condition**	**Cohen^**′**^s d**	**Size (cm^**2**^)**	**Estimate (10^**−10**^)**	**Confidence interval (10**^****−10****^ **)**	
1: right inferior parietal gyrus	250–400	0.012	HP>LP	|HP|<|LP|	0.080	5.10	9.26	[6.11	42.46]
2: left inferior parietal gyrus	550–950	0.020	HP>LP	|HP|>|LP|	0.074	5.72	13.23	[5.88	57.82]
3: left postcentral gyrus	550–950	0.019	HP>LP	|HP|>|LP|	0.075	6.32	11.46	[5.64	50.62]
4: left SFG	550–850	0.023	HP<LP	|HP|<|LP|	0.072	6.10	15.11	[−64.80	−5.48]
5: left inferior precentral gyrus	600–700	0.021	HP<LP	|HP|<|LP|	0.073	5.49	16.69	[−72.41	−6.89]

HP, High potency; LP, Low potency.

### Effect of Valence: Negative vs. Positive Words

The effect of valence in the temporal domain was significant starting from 200 ms until around 500 ms (results shown in Figure 8, and Table 5). This effect starts slightly earlier compared to some previous studies, where the effect was observed starting from 250 to 290 ms (Palazova et al., 2013; Imbir et al., 2016). This could be due to the used task, as in both cited studies a lexical decision task was adopted instead of a categorization task, as in our case, which calls upon semantic processing. One comprehensive overview of written emotion word processing using both fMRI and EEG (Citron, 2012) showed that, for the majority of the cited studies that adopted a lexical decision task, a time window of 200–300 ms marked the onset of the effect of emotional content of verbal material (i.e., a larger amplitude for emotionally charged words over neutral words). However, the same review also suggested a later time window of 500–800 ms which we did not observe. In our source localization analysis, we observed the early time window effect in the left lateral occipital cortex, the right occipito-parietal, middle frontal and superior temporal gyrus, and the prefrontal (frontopolar) cortex bihemispherically. The overall effect was therefore more lateralized in the right hemisphere, which is in line with the commonly accepted processing of emotion in the right hemisphere, including fMRI studies with similar paradigms (Kuchinke et al., 2005; Citron, 2012). In the occipital, prefrontal and occipito-parietal areas, similar to previous ERP studies (Kawachi et al., 2011; Palazova et al., 2013), words with high valence evoke a higher activation in early time windows compared to words with low valence. In the processing of valence, unlike other semantic features, the temporal cortex was involved to a considerably lesser degree and mainly only in the right hemisphere (anterior part of superior temporal gyrus). Activation in the middle frontal cortex was confirmed by similar fMRI studies (Schaefer and Rotte, 2010). On the other hand, also unlike other semantic features, the right superior parietal lobule was primarily involved in processing valence specifically during lexical access and semantic processing (250–500 ms). This could be explained by the involvement of the parietal cortex in processing emotionally salient stimuli (Barbaro et al., 2017). It is interesting to note that many studies have suggested valence as an embodied approach toward the neural representation of abstract concepts (Kousta et al., 2011; Vigliocco et al., 2014). Our study shows the same distinct neural patterns to represent both abstract and concrete concepts when correcting for levels of concreteness in the mixed model.

**Table 5:** Statistical results for significance map of Figure 8 (valence).

**Region**	**Time window (ms)**	***P*-value (corrected)**	**Polarity**	**Stronger condition**	**Cohen^**′**^s d**	**Size (cm^**2**^)**	**Estimate (10^**−10**^)**	**Confidence interval (10**^****−10****^ **)**	
1: left lateral occipital gyrus	250–500	0.022	LV>HV	|LV|<|HV|	0.063	9.66	15.36	[3.24	63.55]
2: right occipito-parietal cortex	250–400	0.016	LV>HV	|LV|>|HV|	0.071	6.52	12.54	[5.84	55.07]
3: right superior temporal gyrus	250–400	0.024	LV>HV	|LV|>|HV|	0.067	12.54	16.75	[5.06	62.98]
4: right and left frontopolar cortex	200–350	0.019	LV>HV	|LV|<|HV|	0.070	9.38	12.31	[5.74	53.21]
5: right middle frontal gyrus	250–400	0.02	LV<HV	|LV|>|HV|	−0.060	9.09	14.34	[−56.95	−0.15]
6: right superior parietal gyrus	300–400	0.015	LV<HV	|LV|<|HV|	−0.074	8.68	7.80	[−35.36	−4.75]

LV, Low Valence; HV, High Valence.

## General Discussion

In order to shed light on the possible neural correlates of semantic category representation, we sat out to chart the networks whose activity differs when processing different semantic features. When evaluating the processing of semantic features, mainly techniques with either good temporal (ERP) or spatial (fMRI) resolution have been adopted, thus addressing either the *when* or *where* questions. We believe that high-density EEG with source localization yields the best of both worlds as it combines spatial with temporal resolution. To this end, we have developed a mass-univariate analysis technique in the source domain using a mixed linear effect model.

To summarize our results, a few regions seem to be playing an important role in processing semantic features. First, we identified the left inferior frontal lobe, which is in line with the increased fMRI activation observed in the lateral inferior prefrontal cortex during deep semantic encoding (Demb et al., 1995; Bookheimer, 2002), from which the authors argued that it might play a central executive role in retrieving semantic information. Indeed, the activity of the inferior frontal lobe seems to differ between different semantic features such as abstractness and activity. In agreement with clinical observations, early functional brain imaging studies of semantic processing revealed activity in broad regions of the left prefrontal, parietal and posterior temporal lobes, commonly including ventral and lateral regions of the temporal cortex (Martin and Chao, 2015). Studies have shown that the ventral and lateral regions of the posterior temporal cortex can be differently engaged in semantic processing, depending on the type of information retrieved. This is consistent with the results from our study, where the temporal cortex seems to be prominently active when processing various semantic features.

On the same note, we believe that the role of the right hemisphere in word processing should be more acknowledged since, in the current study, we found a number of brain areas, in particular in the right temporal cortex, that were largely involved in processing a number of semantic features (i.e., abstractness and valence). This activation was mainly present during lexical access and semantic processing (300–600 ms), providing evidence of the role of the right hemisphere in the semantic processing of a word, as previously reported using mono-hemispheric stimulation studies (Coulson et al., 2005). Finally, certain brain areas participate in the processing of several word features. For instance, the involvement of fronto-temporal areas in our study, similar to some previous investigations (Lau et al., 2008; Brouwer and Hoeks, 2013), was observed in the processing of most features-of-interest. However, it is worth noting that none of the listed areas participated in the processing of all features.

Processing abstract and concrete words involves partially overlapping brain networks. The larger engagement of the left hemisphere over the right when processing abstract words seems to be in favor of the dual coding theory (Binder et al., 2005), but overall we did not observe a consistently larger activation of the right hemisphere for concrete words and cannot support this theory exclusively. When considering the size of the differences, the total area with a stronger activation for abstract words was twice the amount in the opposite condition, and this ratio increased to quadruple the original amount when taking into account only the approximate time window for semantic processing (Table 4). These findings suggest a richer network of brain activation for processing abstract concepts. To the best of our knowledge, we are the first to demonstrate this, and we believe the richer activation can explain the behavioral effect of concreteness, since a larger network would imply more effort and therefore a longer response time for abstract concepts. Since abstract words depend on a looser set of associated knowledge than concrete words, previous research (Binder et al., 2005) has speculated this would transform into stronger activation for concrete words. However, stronger associated knowledge would imply less processing effort. Other studies have also suggested a more extensive spatial distribution in the absence of strong semantic context during single word processing (Wang et al., 2013). Therefore, we believe our result of stronger activation for abstract concepts to be in support of the context availability theory. In the end, our results do not entirely support one single theory over another but combine aspects of both.

The semantic dimensions defined by Osgood show a distributed network of differences. In the current study, dimensions of valence and potency had a higher effect size and occurred earlier in the processing chain compared to activity. This points in the direction of a higher biological value for valence and potency over activity, as their evaluation does not require language since we have evolved to react to more biologically significant stimuli to preserve our lives (Imbir et al., 2016). As such, these dimensions could indeed serve as the necessary semantic space to describe both abstract and concrete concepts. Though we did include concreteness as a random factor in our model, further study is necessary to investigate the interaction between concreteness and these dimensions also to establish whether (which part of) the distributed network is shared by the two dimensions. Additionally, some studies have questioned the distinction of the dimensions and have suggested that there is in fact a high correlation between arousal and valence ratings, which we did not find in our database. Other studies have reported that the self-assessment ratings of potency (as we used in our study) diverge from the original semantic differential scale suggested by Osgood since subjects are rating their own feeling of dominance rather than the dominant meaning of the word (Bradley and Lang, 1994). As for the Moors et al. database we used, the dominant meaning of the word was rated (Moors et al., 2013). Since the correlations between dimensions were also reported, we could verify them for our dataset. We found a weak but significant positive correlation between potency and arousal. To investigate the possible implications of such interactions between these dimensions would constitute a study on its own and is beyond the scope of the present study. Regardless, we do not believe the outcome would undermine our hypothesis, since our purpose was to unveil the spatio-temporal activation patterns for individual Osgood dimensions when processing abstract and concrete words.

On a final note, in EEG source reconstruction two mainstream techniques are used: the equivalent current dipole modeling (parametric) and distributed source models (nonparametric, Grech et al., 2008). The former requires prior specification of the number of neural sources and will solve the inverse solution to find the location and orientation for these sources. The latter assumes the entire cortical surface (or brain volume) to consist of fixed source locations and will solve the issue of their amplitude, but these sources are by no means assumed to be independent and can therefore not be regarded as different neural generators. Since we had no prior knowledge of where to expect our effect (as language processes generally involves a large area of activity; Price, 2015), we used distributed source analysis instead of equivalent current dipoles. However, since we did not perform any analysis to estimate how many sources are interacting, we do not claim that differences in source localization are due to different neural generators, for example both areas located in the inferior parietal lobe of the left hemisphere for potency are likely to originate from the same generator.

## Limitation and Future Study

One limitation of our study was that the solution space for source localization was confined to the cortical surface. This prevented us from investigating activity from deeper structures. Several studies suggested that such activity could be detected from EEG source reconstruction (Attal et al., 2009; Cebolla et al., 2016). Admittedly, the results of our statistical analysis also included medial areas of the cerebral cortex such as the frontopolar gyrus and gyrus rectus, located in the frontal part of the interhemispheric fissure (mainly abstract words, see Supplementary Material in Appendix B, for detailed representation). However, since the contribution of these sources to EEG scalp activity is still under much debate (Attal et al., 2012), we did not include these results in the main manuscript. In addition, because our head model is confined to the cortical surface, it is possible that the activity we observe does in fact originate from subcortical regions, forcedly mapped onto the most nearby cortical surface. To understand where this activation truly comes from, future studies would be necessary to perform source localization on the entire cortical volume, including deeper structures such as the thalamus and cerebellum.

## Conclusion

The purpose of our study was to demonstrate differences in source activation when processing abstract and concrete words also when scoring extremally along the valence, arousal, and potency semantic dimensions defined by Osgood. Our results show that each dimension has a distinct spatio-temporal pattern of activation for the low and high values of a given dimension when correcting for the concreteness of the word. These results are promising in that they could indeed provide evidence for grounding both abstract and concrete words in the same underlying neural system and, in this way, pave the way toward a unified theory on semantic category representation. Since each EEG electrode records a mix of activity from multiple sources (Nunez and Srinivasan, 2006), we believe that the un-mixing performed by source localization can reveal effects previously unnoticed. With the current study, we showed that different neural populations respond to different semantic dimensions. Here, we studied the temporal and spatial dynamics of processing the semantic features of a word. We did not consider words as plain linguistic entities, but rather as complex combinations of lexical and semantic characteristics. Given that our source localization technique allowed us to identify the brain areas involved in semantic feature processing during different stages of word processing, we are in a position to update or alter existing models of spatio-temporal dynamics of word processing.

## Author Contributions

MF conceived and conducted the experiments and performed the analysis. EK, MF, and MMVH participated equally in writing and reviewing the manuscript.

### Conflict of Interest Statement

The authors declare that the research was conducted in the absence of any commercial or financial relationships that could be construed as a potential conflict of interest.
