# Supplementary material for: Semantic Features Reveal Different Networks During Word Processing: An EEG Source Localization Study
Source: Front Hum Neurosci. 2018 Dec 13;12:503. doi: 10.3389/fnhum.2018.00503 (PMC6300518; doi:10.3389/fnhum.2018.00503)
Supplement: Supplementary file 1 [file Presentation_1.pdf]

Supplementary Material

Semantic features reveal different networks during word processing: an EEG source localization study

Mansoureh Fahimi Hnazaee*, Elvira Khachatryan, Marc M. Van Huller

*** Correspondence:** Mansoureh Fahimi Hnazaee: mansoureh.fahimihnazaee@kuleuven.be

Appendix A

Supplementary Table 1: Word Database

Abstract	Concrete		High Activity	Low Activity	High Potency	Low Potency	High Valence	Low Valence
boosheid	hamer	haring	boosheid	roze	boosheid	roze	pauw	boosheid
droefheid	zaag	schol	adelaar	rijst	zwart	ezel	roze	vlo
vreugde	tang	ansjovis	joggen	zwart	adelaar	vlo	giraf	zaag
blijdschap	bijl	zwaardvis	kussen	brood	zaag	mier	brood	afgunst
walging	spijker	goudvis	specht	ruit	vreugde	kalkoen	adelaar	vlieg
afkeer	beitel	piranha	mier	groen	blijdschap	krekel	joggen	mug
afgunst	schroef	inktvis	vreugde	bank	leeuw	schaap	kussen	jaloezie
jaloezie	werktuig	tonijn	blijdschap	sofa	olifant	vlieg	groen	luis
wanhoop	ploeg	paling	leeuw	vierkant	valk	mug	sofa	grijs
vrijheid	wortel	frieten	mug	bruin	walging	vlinder	vreugde	walging

democratie	tomaat	pasta	jaloezie	schaap	hamer	rups	zebra	bijl
communisme	komkommer	spaghetti	valk	soep	tijger	luis	soep	kakkerlak
moral	bloemkool	soep	walging	kegel	wolf	tomaat	blijdschap	boksen
idealisme	selderij	koekjes	tijger	biet	bijl	beige	leeuw	wesp
religie	spinazie	boterham	wolf	matras	ploeg	konijn	olifant	droefheid
realisme	spruitjes	rijst	bijl	rups	boksen	duif	matras	wanhoop
justitie	uien	fruit	ploeg	haard	beer	eend	vlinder	spin
bijgeloof	erwten	groenten	tennis	laken	beitel	forel	haard	worm
	aubergine	brood	boksen	blauw	rood	droefheid	pasta	gier
	biet	snoep	schaatsen	grijs	spijker	wanhoop	tomaat	afkeer
	knoflook	gebak	wesp	tomaat	vrijheid	worm	kachel	
	vlieg	kanarie	beer	beige		kever	tennis	
	mug	duif	spin	kachel		muis	konijn	
	mier	kraai	zwemmen	cirkel			schaatsen	
	wesp	adelaar	vrijheid	tafel			zwaan	
	spin	specht	voetbal	droefheid			zwaluw	
	kever	parkiet	basketbal	gebak			hert	
	vlinder	zwaluw		worm			gebak	
	kakkerlak	eend		stoel			zwemmen	
	worm	gier		ansjovis			snoep	
	hommel	valk		zetel			zetel	
	vlo	pauw		kruk			vrijheid	
	krekel	zwaan		lamp			fruit	
	luis	kalkoen		wortel			basketbal	
	bloedzuiger	fazant		fruit				
	rups	haan		raam				
	stoel	leeuw						
	tafel	tijger						
	zetel	olifant						
	bureau	giraf						

kapstok	zebra
matras	ezel
kussen	schaap
laken	beer
lamp	wolf
raam	kameel
kruk	hert
sofa	varken
bank	lama
haard	muis
kachel	konijn
zalm	
kabeljauw	
forel	

Appendix B

Supplementary Figure 1: Deep regions in Abstractness

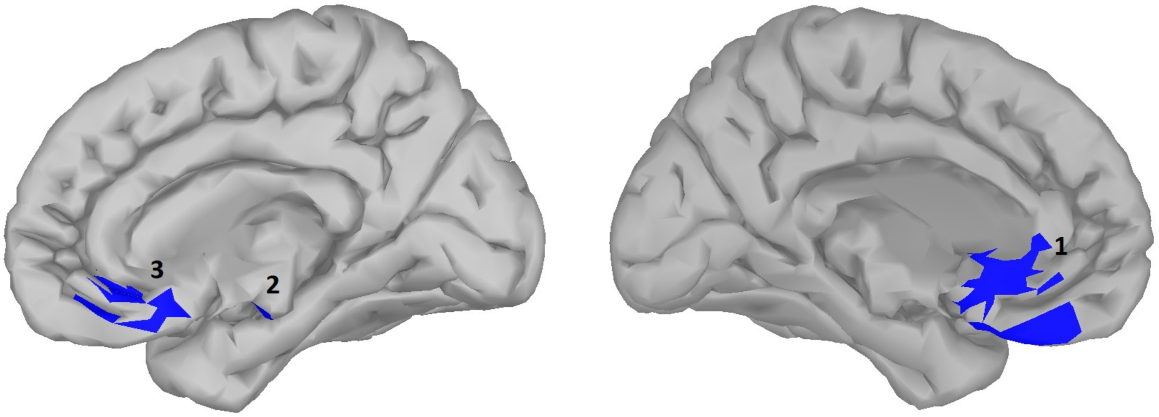

Supplementary Table 2

Region	Time Window (ms)	P value (corrected)	Polarity	Stronger Condition	Cohen's d	Size (cm^2)	Estimate (10^{-10} A.m)	Confidence Interval (10^{-10} A.m)

1	50-350	0,027	A>C	|A|>|C|	0,0362	12.01	4.56	[1.30	19.21]
2	300-650	0,016	A>C	|A|>|C|	0,0401	4.80	9.81	[1.37	43.53]
3	550-700	0,018	A<C	|A|>|C|	0,035	8.34	8.84	[-36.46	-1.76]
